# Ultrasound and clinicopathological characteristics-based model for prediction of pathologic response to neoadjuvant chemotherapy in HER2-positive breast cancer: a case–control study

**DOI:** 10.1007/s10549-023-07057-0

**Published:** 2023-08-28

**Authors:** Lin Sui, Yuqi Yan, Tian Jiang, Di Ou, Chen Chen, Min Lai, Chen Ni, Xi Zhu, Liping Wang, Chen Yang, Wei Li, Jincao Yao, Dong Xu

**Affiliations:** 1 Postgraduate training base Alliance of Wenzhou Medical University (Zhejiang Cancer Hospital), Hangzhou, China; 2 Department of Diagnostic Ultrasound Imaging & Interventional Therapy, Zhejiang Cancer Hospital, Hangzhou Institute of Medicine (HIM), Chinese Academy of Sciences, Hangzhou, China; 3 Key Laboratory of Head & Neck Cancer Translational Research of Zhejiang Province, Hangzhou, China; 4 Zhejiang Provincial Research Center for Cancer Intelligent Diagnosis and Molecular Technology, Hangzhou, China; 5 Wenling Big Data and Artificial Intelligence Institute in Medicine, Taizhou, China; 6 Taizhou Key Laboratory of Minimally Invasive Interventional Therapy & Artificial IntelligenceTaizhou Branch of Zhejiang Cancer Hospital(Taizhou Cancer Hospital), Taizhou, China; 7 Graduate School, Wannan Medical College, Wuhu, China; 8 The Second Clinical Medical College of Zhejiang Chinese Medical University, Hangzhou, China

**Keywords:** HER2-positive breast cancer, Neoadjuvant chemotherapy, Pathologic complete response, Ultrasound

## Abstract

**Background:**

The objective of this study was to develop a model combining ultrasound (US) and clinicopathological characteristics to predict the pathologic response to neoadjuvant chemotherapy (NACT) in human epidermal growth factor receptor 2 (HER2)-positive breast cancer.

**Materials and methods:**

This is a retrospective study that included 248 patients with HER2-positive breast cancer who underwent NACT from March 2018 to March 2022. US and clinicopathological characteristics were collected from all patients in this study, and characteristics obtained using univariate analysis at *p* < 0.1 were subjected to multivariate analysis and then the conventional US and clinicopathological characteristics independently associated with pathologic complete response (pCR) from the analysis were used to develop US models, clinicopathological models, and their combined models by the area under the receiver operating characteristic (ROC) curve (AUC), accuracy, sensitivity, and specificity to assess their predictive efficacy.

**Results:**

The combined model had an AUC of 0.808, a sensitivity of 88.72%, a specificity of 60.87%, and an accuracy of 75.81% in predicting pCR of HER2-positive breast cancer after NACT, which was significantly better than the clinicopathological model (AUC = 0.656) and the US model (AUC = 0.769). In addition, six characteristics were screened as independent predictors, namely the Clinical T stage, Clinical N stage, PR status, posterior acoustic, margin, and calcification.

**Conclusion:**

The conventional US combined with clinicopathological characteristics to construct a combined model has a good diagnostic effect in predicting pCR in HER2-positive breast cancer and is expected to be a useful tool to assist clinicians in effectively determining the efficacy of NACT in HER2-positive breast cancer patients.

## Introduction

Breast cancer is the most prevalent cancer among women and is a heterogeneous solid tumor with complex genetic and molecular variations [1–3]. There are 5 main types of treatment for breast cancer: surgery, chemotherapy, radiotherapy, endocrine therapy, and targeted therapy, of which surgery is one of the principal treatment options [4]. However, for some patients who are not suitable for direct surgery due to large breast cancer foci, extensive metastases, and distant metastases or a strong desire for breast conservation, neoadjuvant chemotherapy (NACT) is first administered to these patients to reduce their clinical stage, increase their breast conservation rate, and reduce the rate of axillary surgery [5, 6]. Meanwhile, NACT provides an opportunity to monitor tumor response and assess drug efficacy in real time [7]. Human epidermal growth factor receptor 2 (HER2)-positive breast cancer accounts for 15%–20% of all breast cancers and has an increased risk of local recurrence and metastasis and a poor overall prognosis [8]. Previous studies have shown that HER2-positive breast cancer has a high sensitivity to NACT and its prognosis is significantly improved [9]. Therefore, NACT has become the standard of care for HER2-positive breast cancer [10]. Pathologic complete response (pCR) is a key indicator to assess the efficacy of NACT and can be employed as an early surrogate endpoint to predict patients with higher disease-free survival (DFS) and overall survival (OS) after NACT [11]. Research [12] has shown that HER2-positive breast cancer patients have better long-term benefits after reaching pCR. However, due to the heterogeneity of HER-2-positive breast cancers, the response to NACT varies, with the probability of achieving pCR ranging from 20 to 80%, and some patients still fail to achieve pCR [13]. Therefore, early prediction of whether pCR will be achieved after NACT treatment is of great clinical significance for HER2-positive breast cancer patients and can assist clinicians to adjust treatment regimens as early as possible It can assist clinicians in making early adjustments to treatment regimens, reduce unnecessary toxic effects of chemotherapy, increase pCR rates, and improve patient prognosis.

NACT is a long-term treatment process during which tumor changes occur owing to fibrosis, fragmentation, and/or necrosis, so imaging allows for non-invasive monitoring of treatment response during this time and serves to predict which patients will achieve pCR early in the course of treatment [8, 14]. This method has served in many previous studies to monitor the response of breast cancer after early treatment with NACT. A study [15] explored the correlation between mammography density and the pathologic response to NACT in breast cancer. However, prolonged exposure to X-rays may represent a health risk to the patient, due to their ionizing nature. A retrospective study [16] including 296 HER2-positive breast cancer patients who underwent NACT showed that the use of radiation-free MRI can effectively forecast pCR after NACT in HER2-positive breast cancer, particularly in the hormone receptor (HR)-negative subtype. However, this imaging method still has limitations, such as the long and expensive examination time and the difficulty of performing multiple repeat MRI examinations in a short period. Ultrasound (US) is now widely used and has the advantages of being reproducible, non-ionizing, and well-tolerated by patients to monitor changes in mass size, shape, elasticity, and blood flow. Several previous studies have evaluated the accuracy of the US in identifying pCR in breast cancer patients undergoing NACT. However, only a small number of HER2-positive cases were enrolled in these studies [17, 18]. Therefore, 248 HER2-positive patients after receiving complete NACT were included in this study. The efficacy of pCR in the primary focus of HER2-positive breast cancer patients after NACT was predicted by exploring the US characteristics of the focus before NACT treatment and clinical pathologic characteristics.

## Material and methods

### Patients

**Fig. 1:**
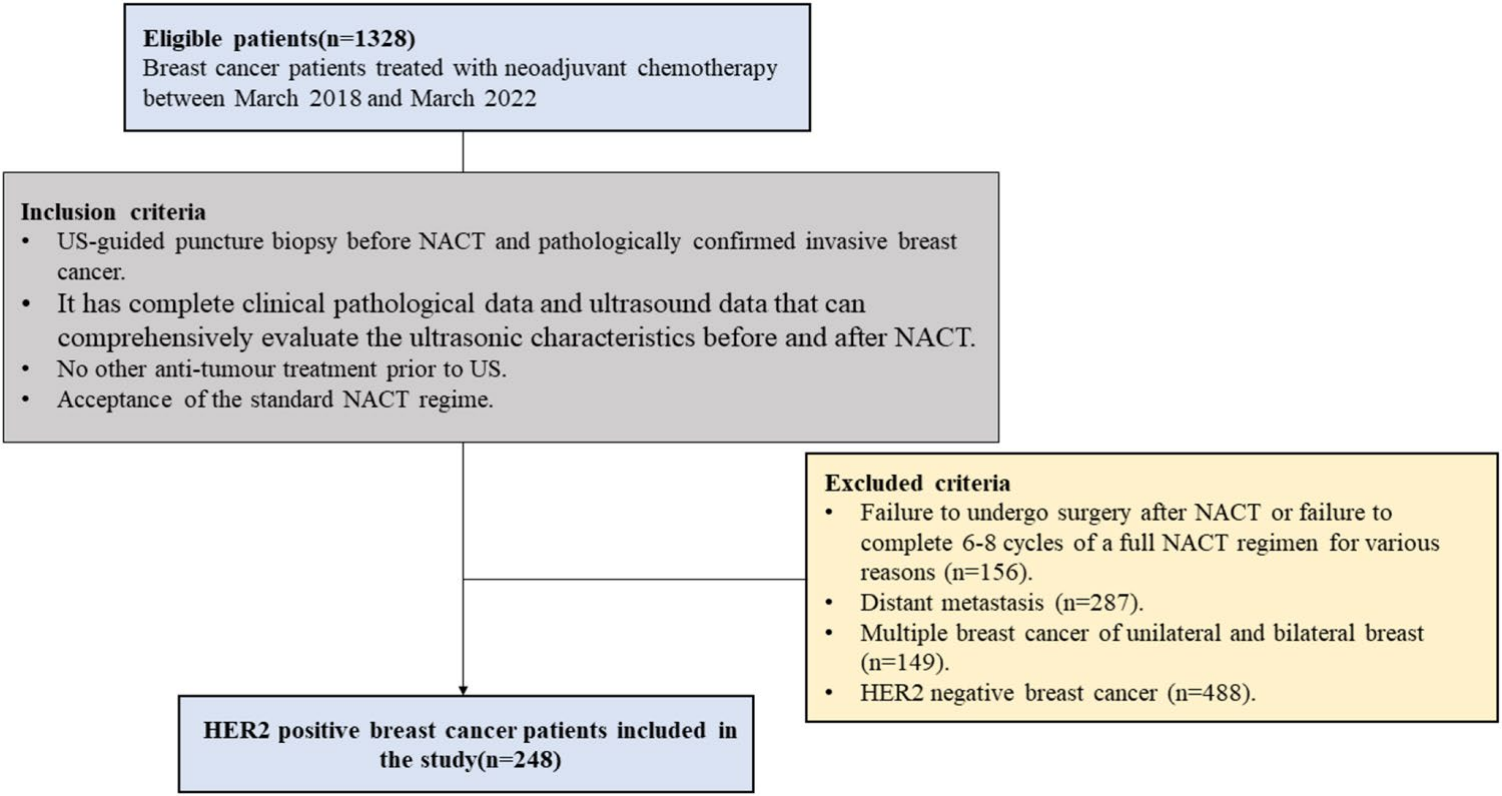
Flowchart of HER2-positive patients’ enrollment process

This study retrospectively collected cases of 1328 female breast cancer patients who received NACT at Zhejiang Cancer Hospital from March 2018 to March 2022. All patients underwent US examination and US-guided puncture biopsy before NACT. The inclusion criteria were as follows: ①US-guided puncture biopsy before NACT and pathologically confirmed invasive breast cancer. ②It has complete clinical pathologic data and US data that can comprehensively evaluate the ultrasonic characteristics before and after NACT. ③No other anti-tumor treatment before the US. ④Acceptance of the standard NACT regime. The exclusion criteria were as follows: ①Failure to undergo surgery after NACT or failure to complete 6–8 cycles of a full NACT regimen for various reasons (*n* = 156). ②Distant metastasis (*n *= 287). ③Multiple breast cancer of the unilateral and bilateral breast (*n* = 149). ④HER2 negative breast cancer (*n* = 488). Finally, a total of 248 HER2-positive patients were included in this study (Fig. 1). The study design and protocol were approved by the Ethics Committee of the Zhejiang Cancer Hospital Review Board (IRB-2023-125) and the requirement for written informed consent was waived.

### Ultrasonographic data collection

All patients had US examinations before and after NACT using a GE Logiq E9 ultrasonic instrument (General Electric Healthcare, Milwaukee) with a high-resolution linear probe (ML6-15) and a Philips iU22 ultrasonic instrument (Philips Healthcare, the Netherlands) with a high-frequency linear probe (L12-5). In this study, considering that different doctors’ US reports may have different interpretations of the same characteristics, three doctors with more than 5 years of experience were selected to interpret the corresponding US maps stored in the database. If there were differences, an agreement was reached after discussion. ① [19] Routine US features of breast lesions include maximum diameter (mm), echogenicity (hypoechoic or non-hypoechoic), shape (regular or irregular), lateral shadow (present or absent), posterior acoustic (attenuated or non-attenuated), margins (spiculation or non-spiculation), boundary (clear or blurred), and calcification (present or absent). ② The blood flow was graded into three types (grade 1, 2, and 3) based on its richness with Adeler classification method [20], which defines blood flow classification: grade 0 to 1 as low score and grade 2 to 3 as high score. ③ Elasticity score: These are divided into 0 to 5 grades according to the different colors of the elastogram [21], where a graded Elasticity score is defined: 1 to 3 as a low score and 4 to 5 as a high score.

### Histopathology analysis

For all patients, an US-guided puncture biopsy of the target breast tumor was performed and IHC indices were measured before NACT. We collected the estrogen receptor (ER), progesterone receptor (PR), HER2, and Ki67 index status of the pathology reports. ① The critical level of Ki67 is 30% [22]. ② ER- and PR-positive definition: IHC staining ≥ 1% positively stained tumor cells [23]. ③ HER2 positive defined as IHC 3 + or IHC 2 + and amplified by fluorescence in situ hybridization (FISH); HER2 negative defined as IHC 0 or IHC 1 + or IHC 2 + and FISH negative [24]. ④ Molecular subtypes were split into HR positive and HR negative. The pCR status of each target tumor was determined by surgical pathology results within 1 month after the completion of the full cycle of NACT. The pCR status is evaluated based on the residual tumor of the primary breast lesion in postoperative pathology. The cellular profile of the resected tumor specimens was compared to pre-chemotherapy according to the MP grading system [25]: Grade 1: No change or some alteration to individual malignant cells but no reduction in overall cellularity; Grade 2: A minor loss of tumor cells < 30%; Grade 3: Between an estimated 30% and 90% reduction in tumor cells; Grade 4: A marked disappearance of tumor cells, more than 90% loss of tumor cells; and Grade 5: No malignant cells identifiable in sections from the site of the tumor, only vascular fibroelastotic stroma remains often containing macrophages. However, ductal carcinoma in situ (DCIS) may be present. Of these, grades 1–4 are considered non-pathologic complete response (non-pCR) and grade 5 is considered pCR.

### Neoadjuvant chemotherapy

The NACT regimen follows the National Comprehensive Cancer Network (NCCN) and China Anti-Cancer Association (CACA) guidelines. HER2-targeted therapy (trastuzumab in combination with or without pertuzumab) is added to chemotherapy for patients with HER2-positive breast cancer. Treatment cycles of 6 or 8 course of 21 days each.

### Clinicopathological data

Clinicopathological information was obtained from the patient’s medical records. Clinical data included age, menopausal status, clinical TNM stage, NACT regimen, NACT cycle, and surgical approach. Histopathological findings included tumor type, ER status, PR status, HER2 status, Ki67 index, and pCR status.

### Statistical analysis

For all statistical analyses, SPSS (version 25.0) and R (version 4.2.1) were used. Continuous variables were expressed as mean ± standard deviation (SD) or median M (interquartile range IQR), t test, or Mann–Whitney *U* test for comparisons between two groups. Categorical information is expressed as instances (%) and comparisons between two groups were made using the *χ*2 test or Fisher’s exact test. Statistical significance was defined as a two-tailed *p* < 0.05.

All the clinicopathological and conventional US characteristics associated with pCR were first assessed using univariate analysis and then variables with *p* < 0.1 in the univariate analysis were entered into a multivariate logistic regression analysis to determine independent correlations between the above characteristics and pCR. Subsequently, a combined model was developed by integrating conventional US and clinicopathological characteristics using multivariate logistic regression analysis. The area under a receiver operating characteristic (ROC) curve (AUC) was utilized to compare the predictive effect of the conventional US model, the clinicopathology model, and the combined model on pCR status. An AUC value greater than 0.8 is considered acceptable [26].

## Results

### Patient characteristics

A total of 248 patients with HER2-positive breast cancer (mean age ± standard deviation, 51.81 years ± 9.46; range, 24–76 years) who received the full NACT regimen were included in this study, 146 postmenopausal patients (58.9%) and 102 premenopausal patients (41.1%). All patients had pathologically confirmed invasive carcinoma, including 232 (93.5%) invasive ductal carcinoma (IDC) and 16 (6.5%) non-IDC. All patients underwent breast and axillary surgery after NACT, with 20 patients undergoing breast-conserving surgery and 228 mastectomies (8.1% and 91.9%, respectively). Post-operative pathology showed a breast pCR rate of 53.6% (133/248) and a non-pCR rate of 46.4% (115/248) (Table 1).

**Table 1:** Clinicopathological characteristics of HER2-positive patients

Characteristics	pCR (*n* = 133)	non-pCR (*n* = 115)	*p* value
Age*(mean* ± *SD)*	51.71 ± 8.95	51.91 ± 10.06	0.869
Menopausal status			0.660
Postmenopausal	80 (60.2%)	66 (57.4%)	
Premenopausal	53 (39.8%)	49 (42.6%)	
Location of tumor			0.185
Left breast	85 (63.9%)	64 (55.7%)	
Right breast	48 (36.1%)	51 (44.3%)	
Clinical T stage			0.013
T1	22 (16.5%)	8 (7.0%)	
T2	92 (69.2%)	77 (67.0%)	
T3	16 (12.0%)	20 (17.4%)	
T4	3 (2.3%)	10 (8.7%)	
Clinical N stage			0.020
N0	14 (10.5%)	11 (9.6%)	
N1	87 (65.4%)	59 (51.3%)	
N2	18 (13.5%)	16 (13.9%)	
N3	14 (10.5%)	29 (25.2%)	
Histological type			0.828
IDC	124 (93.2%)	108 (93.9%)	
Non-IDC	9 (6.8%)	7 (6.1%)	
ER status			0.313
Negative	71 (53.4%)	54 (47.0%)	
Positive	62 (46.6%)	61 (53.0%)	
PR status			0.010
Negative	100 (75.2%)	69 (60.0%)	
Positive	33 (24.8%)	46 (40.0%)	
Ki67			0.464
≤ 30%	54 (40.6%)	52 (45.2%	
> 30%	79 (59.4%)	63 (54.8%)	
Molecular subtype			0.304
HR negative	70 (52.6%)	53 (46.1%)	
HR positive	63 (47.4%)	62 (53.9%)	
NACT regimen			0.002
Taxane based	85 (63.9%)	51 (44.3%)	
Anthracycline and taxane based	48 (36.1%)	64 (55.7%)	
Anti-HER2 therapy			< 0.001
H	29 (21.8%)	49 (42.6%)	
HP	104 (78.2%)	66 (57.4%)	
Tumor surgery type			0.898
Breast-conserving surgery	11 (8.3%)	9 (7.8%)	
Mastectomy	122 (91.7%)	106 (92.2%)	

*HER2* human epidermal growth factor receptor 2; *SD* standard deviation; *IDC* invasive ductal carcinoma; *ER* estrogen receptor; *PR* progesterone receptor; *HR* hormone receptor; *pCR* pathologic complete response; *NACT* neoadjuvant chemotherapy; *H* trastuzumab; *HP* trastuzumab plus pertuzumab

### Univariate analyses

For the univariate analysis of Clinicopathological characteristics (Table 1), HER2-positive patients with pCR exhibited lower clinical T stage (*p* = 0.013), lower clinical N stage (*p* = 0.020), and negative PR compared to non-pCR (*p* = 0.010). In addition, the NACT regimen and Anti-HER2 therapy were significantly correlated with pCR status (all *p* < 0.05). There were no statistically significant differences between the two groups in terms of age, menopausal status, location of the tumor, histological type, and molecular subtype.

For the univariate analysis of breast US characteristics (Table 2), tumor margin (*p* < 0.001), posterior acoustic (*p* < 0.001), calcification (*p* < 0.001), and elasticity score (*p* = 0.001) were significantly correlated with pCR. There was no significant difference between the two groups in terms of maximum diameter, echogenicity, shape, boundary, lateral shadow, and blood flow score.

**Table 2:** Ultrasonographic characteristics of HER2-positive breast cancer before NACT

Characteristics	pCR (*n* = 133)	non-pCR (*n* = 115)	*p* value
Maximum diameter (mm)	29.50 (23.00, 41.00)	33.00 (26.00, 41.00)	0.106
Echogenicity			0.653
Hypoechoic	113 (85.0%)	100 (87.0%)	
Non-hypoechoic	20 (15.0%)	15 (13.0%)	
Shape			0.221
Regular	5 (3.8%)	1 (0.9%)	
Irregular	128 (96.2)	114 (99.1%)	
Boundary			0.425
Clear	17 (12.8%)	11 (9.6%)	
Blurred	116 (87.2%)	104 (90.4%)	
Margin			< 0.001
Spiculation	26 (19.5%)	56 (48.7%)	
Non-spiculation	107 (80.5%)	59 (51.3%)	
Lateral shadow			0.067
Presence	62 (46.6%)	67 (58.3%)	
Absence	71 (53.4%)	48 (41.7%)	
Posterior acoustic			< 0.001
Attenuation	24 (18.0%)	58 (50.4%)	
Non-attenuation	109 (82.0%)	57 (49.6%)	
Calcification			< 0.001
Presence	51 (38.3%)	76 (66.1%)	
Absence	82 (61.7%)	39 (33.9%)	
Blood flow score			0.066
Low score	78 (58.6%)	54 (47.0%)	
High score	55 (41.4%)	61 (53.0%)	
Elasticity score			0.001
Low score	80 (60.2%)	45 (39.1%)	
High score	53 (39.8%)	70 (60.9%)	

*HER2* human epidermal growth factor receptor 2; *NACT* Neoadjuvant chemotherapy; *pCR* pathologic complete response

**Fig. 2:**
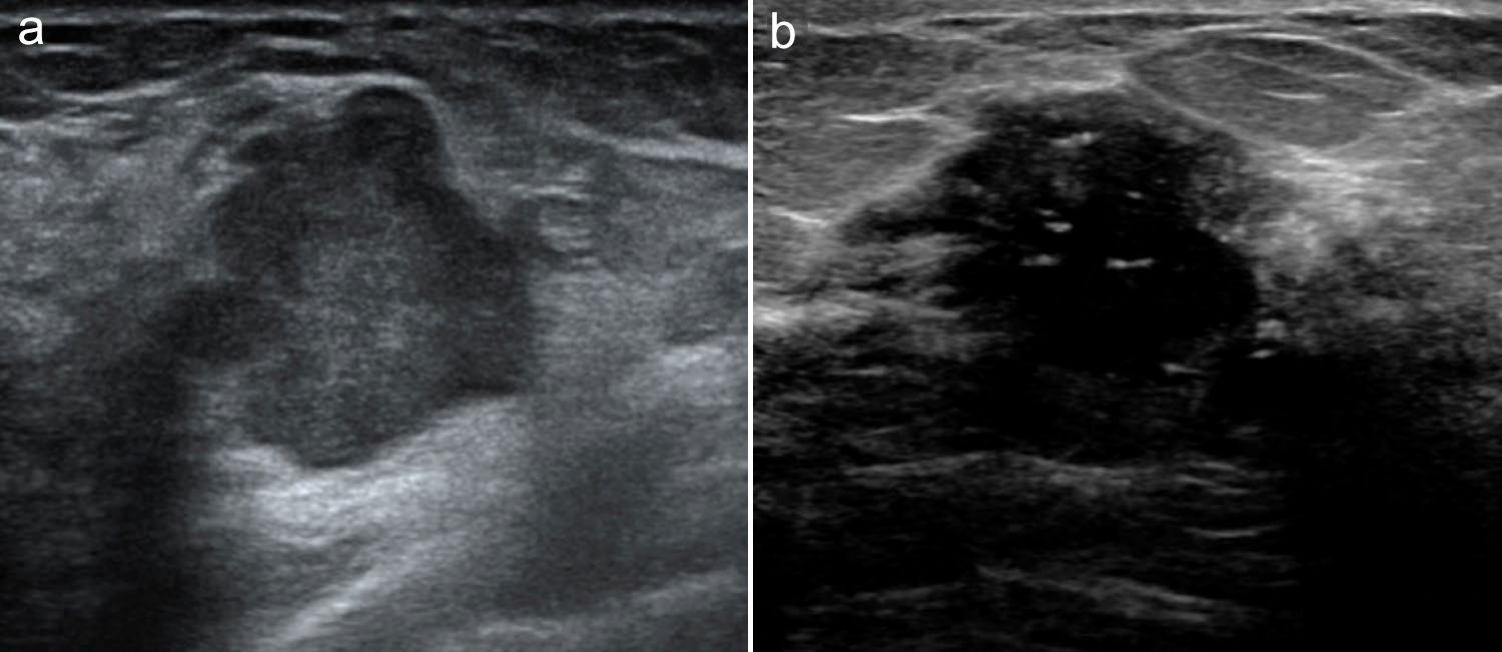
Pre-NACT ultrasound images of two patients with HER2-positive breast cancer who obtained different pathologic responses. **a** Breast ultrasound image of a pCR patient showing blurred nodal margin and posterior acoustic enhancement. **b** Breast ultrasound image of a non-pCR patient showing spiculated margin, internally scattered strong echogenic spots and posterior acoustic attenuation

Figure 2 shows representative pre-NACT breast US images of HER2-positive breast cancer patients in the pCR and non-pCR groups.

### Multivariate analyses

**Fig. 3:**
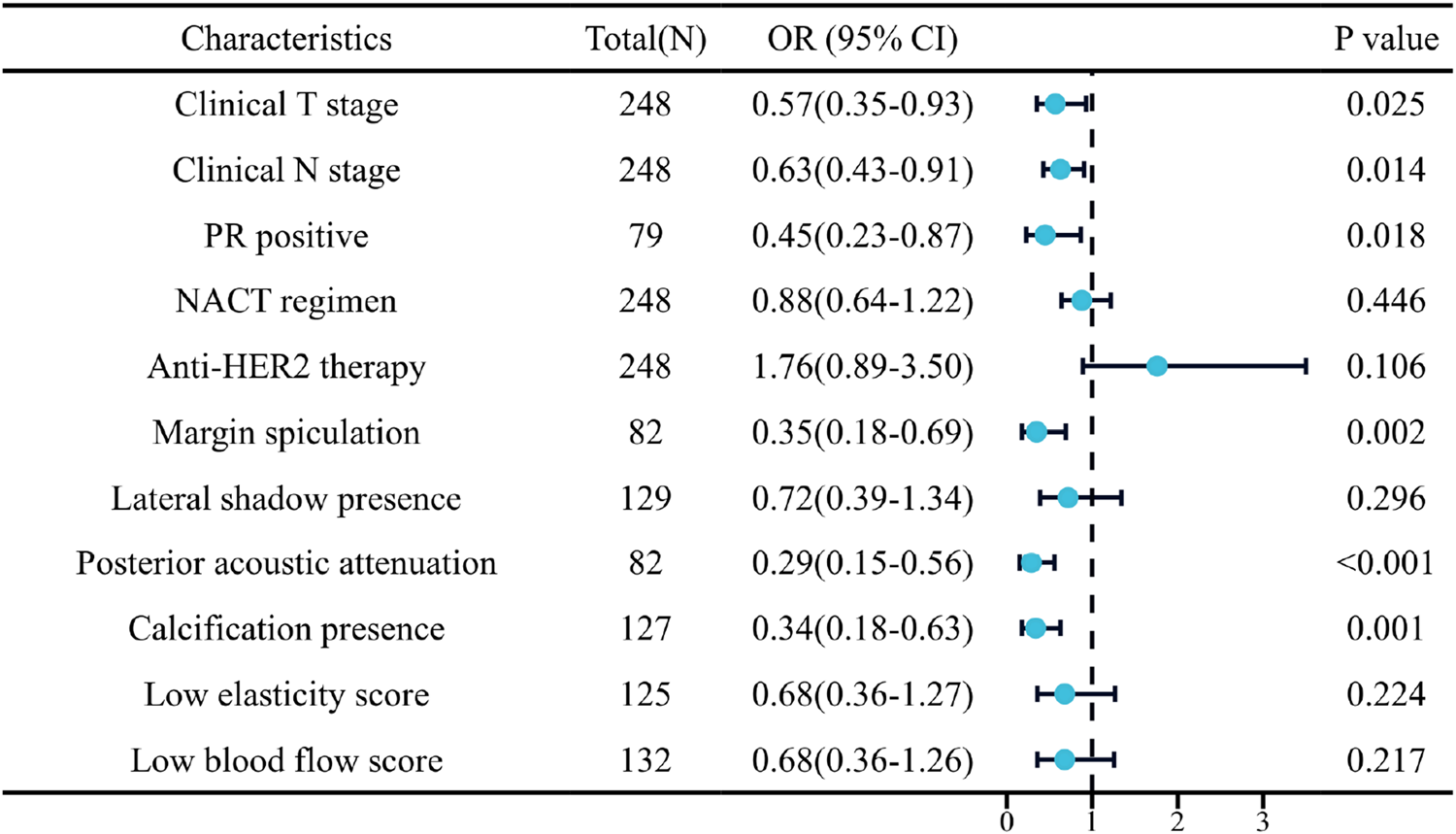
Forest plot for multivariate logistic regression analysis

Variables with p-values less than 0.1 in the univariate analysis were selectively included in the binary logistic regression for multifactorial analysis and the results were found (Fig. 3). Clinical T stage, Clinical N stage, PR, margin, posterior acoustic, and calcification were independent predictors of pCR (all *p* < 0.05). Increasing Clinical T stage grade decreased the pCR rate (OR 0.57; 95% CI 0.35 to 0.93); increasing Clinical N stage grade decreased the pCR rate (OR 0.63; 95% CI 0.43 to 0.91); and PR-positive pCR rate decreased relative to PR negative (OR 0.45. 95% CI 0.23 to 0.87). Compared with non-spiculation, the pCR rate was lower for spiculation at the margin of the breast mass (OR 0.35; 95% CI 0.18 to 0.69); lower for posterior acoustic attenuation compared with non-attenuation (OR 0.29; 95% CI 0.15 to 0.56); and lower for the presence of calcification compared with absence of calcification (OR 0.34; 95% CI 0.18 to 0.63). All of the above factors were hindering factors for pCR.

### Diagnostic performance

**Fig. 4:**
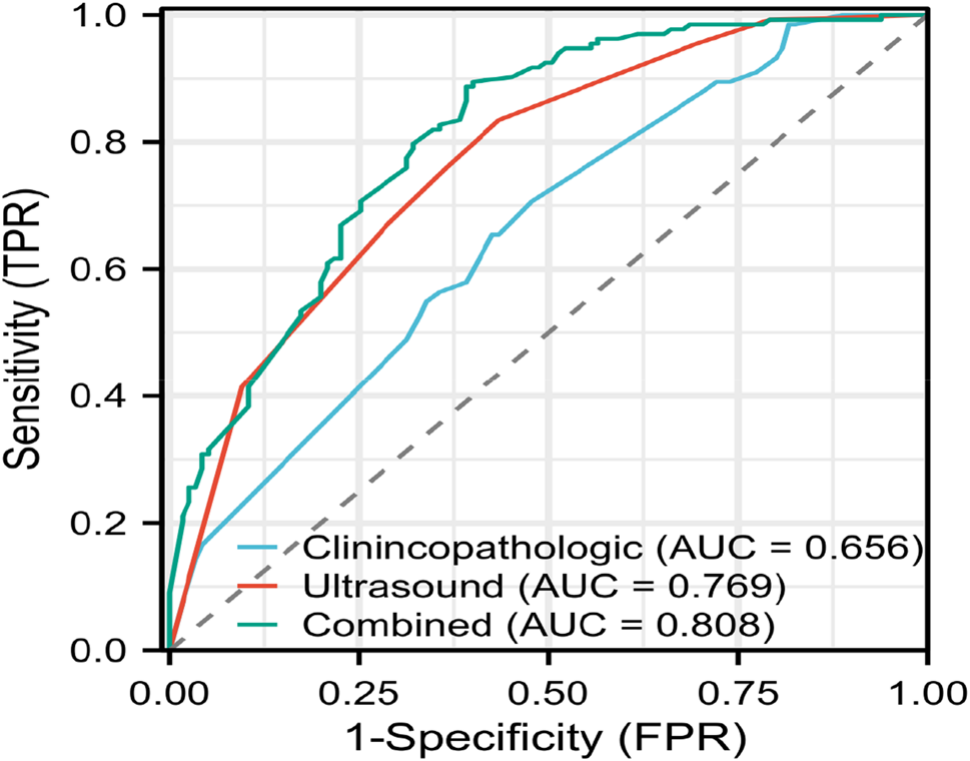
Comparison of ROC curves of the clinicopathological model, ultrasound model, and combined model

ROC curves were drawn to assess the predictive ability of pCR based on clinicopathological characteristics, US characteristics, and combined clinicopathological and US characteristics independently associated with pCR, respectively. Clinicopathological characteristics, including Clinical T stage, Clinical N stage, and PR, were used to construct a clinicopathological model with an AUC of 0.656 and sensitivity, specificity, and accuracy of 70.68%, 52.17%, and 62.10%, respectively. US characteristics including posterior echogenicity, margin, and calcification were used to construct a US model with an AUC of 0.769 and a sensitivity, specificity, and accuracy of 83.46%, 56.52%, and 70.97%, respectively. A combined model was constructed by integrating US and clinicopathology variables independently associated with pCR. When compared with the clinicopathology and US models, the combined model achieved better diagnostic performance with an AUC of 0.808 and improved sensitivity, specificity, and accuracy to 88.72%, 60.87%, and 75.81%, respectively. The AUC of the combined model was significantly better than that of the clinicopathological and US feature models (*p* < 0.001, DeLong’s test) (Fig. 4). Detailed statistical results for all models are presented in Table 3.

**Table 3:** Comparison of the predictive performance of different models

Model	Sensitivity (%)	Specificity (%)	Accuracy (%)	PPV (%)	NPV (%)	FNR (%)	AUC	95%CI	Youden index	Cut-off value	*p* value
Clinicopathological	70.68	52.17	62.10	63.09	60.61	29.32	0.656	0.590–0.723	0.23	0.48	< 0.001
Ultrasound	83.46	56.52	70.97	68.94	74.71	16.54	0.769	0.712–0.827	0.40	0.44	< 0.001
Combined	88.72	60.87	75.81	72.39	82.35	11.28	0.808	0.754–0.861	0.50	0.41	< 0.001

*PPV* positive predictive value; *NPV* negative predictive value; *FNR* false-negative rate

## Discussion

The purpose of this study was to integrate US and clinicopathological characteristics to predict whether patients with HER2-positive breast cancer undergoing NACT would achieve pCR, as US allows for non-invasive and dynamic observation of tumor changes throughout preoperative NACT to allow for timely changes in treatment regimen in case of poor treatment outcome. Therefore, we chose the US to assess breast lesions and to incorporate the clinicopathological characteristics of the lesions. Independent predictors obtained by univariate and multifactorial analyses were used as model variables for the US model (AUC of 0.769) and the clinicopathological model (AUC of 0.656), respectively. To improve the predictive performance of the model, we integrated the US and clinicopathological characteristics to construct a regression model with an AUC of 0.808 for the combined model, which further demonstrated the great clinical application of the combined model we constructed in predicting the early outcome of NACT in HER2-positive breast cancer patients.

HER2 is a transmembrane glycoprotein with receptor tyrosinase activity [27]. In normal cells, the HER2 protein transports growth signals from outside the cell to inside the cell, thus promoting normal growth and division activities. Once HER2 is overexpressed, the cells are stimulated to increase wildly, and the cells become significantly more aggressive and prone to metastasis [28]. Currently, NACT for HER2-positive breast cancer has improved the pCR of patients and significantly improved the prognosis of patients. The American Society of Clinical Oncology (ASCO) guidelines [29] state that trastuzumab, patuximab, and paclitaxel are recommended as first-line therapy for patients with HER2-positive advanced breast cancer. The main mechanism of action of trastuzumab is to inhibit the HER2 homodimer signaling pathway in cancer cells, thereby inhibiting tumor cell growth [30]. Pertuzumab inhibits the formation of both homo- and heterodimers and blocks signaling the source. The combination of the two can play a role in antibody-dependent cell-mediated cytotoxicity (ADCC), which can better mediate immune cells to kill cancer cells [31].The use of NACT has improved the prognosis of HER2-positive breast cancer patients, but some HER2-positive patients still fail to achieve pCR, and considering the side effects of NACT, such as gastrointestinal side effects and cardiotoxicity. Therefore, studies on the assessment of pathologic response and its prediction after NACT in HER2-positive patients are of major clinical importance.

In the present study, the independent predictors of pCR in terms of clinicopathological characteristics were the Clinical T stage, Clinical N stage, and PR status, in line with many previous studies [32–34]. The clinical T stage represents the size of the tumor and the degree of local area infiltration, while the clinical N stage represents the status of the lymph nodes. The lower clinical T stage and clinical N stage in this study had a higher pCR rate. The reason for this analysis is that the higher the two stages, the larger the tumor, the greater the number of lymph node metastases, the higher the degree of invasion, the higher the tumor load, and the more difficult it is for patients to achieve pCR after receiving NACT. In addition, studies have shown that HR expression status was also associated with pCR in HER2-positive breast cancer patients [11]. Our study analyzed the correlation between HR expression and pCR. The results showed that there was no statistical difference between HR-negative and HR-positive groups and ER-negative and ER-positive groups, but PR-negative and PR-positive groups were significantly different and PR negative had a higher pCR rate. Therefore, the correlation between HR status and pCR needs to be explored in further studies. In terms of US characteristics, our results suggest that the posterior echogenicity, margin, and calcification of the mass are all independent predictors of pCR correlation. Previous studies have shown that low-level tumors tend to produce posterior echo attenuation, which refers to a category of neoplastic growths characterized by their relatively limited aggressiveness and potential for malignant progression. These tumors typically exhibit slow growth rates, lower metastatic tendencies, and a generally indolent clinical course compared to high-grade or aggressive malignancies, so it is generally believed that low-level tumors also have poor responses to NACT [35–37]. This could explain the correlation between the absence of attenuation of the posterior acoustic and the pCR can be explained. In addition, we hypothesize that the higher frequency of margin spiculation in low-grade tumors is due to the more frequent pro-connective tissue proliferation response in low grade tumors [38]. Therefore, a higher pCR rate for non-spiculation at the tumor margin can also be justified. Mazari et al. [39] specifically evaluated 111 HER2-positive breast cancer patients, of whom 72 (64.9%) had calcification and 39 (35.1%) had no calcification and showed that the pCR rate was lower in the calcified group than in the non-calcified group (29.2% vs 41%), consistent with our conclusion that calcification was a negative predictor of pCR and that breast cancer patients with calcification were less likely to pCR. Most previous studies based on US have achieved good prediction results for pCR prediction of breast cancer receiving NACT [40–42], but they are studies on all molecular subtyped breast cancers, and lack of comprehensive evaluation of each molecular subtyped breast cancer, especially for HER2-positive breast cancer with poor prognosis. This kind of breast cancer has a high pCR rate after receiving NACT, but some still failed to obtain pCR. Therefore, this study aims to predict the pCR of HER2-positive breast cancer, so as to assist the clinical accurate evaluation of its curative effect and prognosis. Cui et al. [42] predicted pCR by analyzing the US characteristics of 282 patients with advanced breast cancer who underwent NACT. The results showed that the change of mass size, posterior acoustic mode, and elasticity score were independent predictors of pCR. Among them, the posterior acoustic mode was consistent with the conclusion of this study. In addition, Cui et al. [42] constructed a US prediction model based on nomogram on this basis, with an AUC of 0.79, and achieved good prediction performance. However, this study included all breast cancers for analysis, while this study only targeted at HER2-positive breast cancer, and achieved better results in terms of prediction effect. By integrating US characteristics with clinicopathological characteristics to build a combined model, the AUC of this study was 0.808. This fully reflects the great clinical application value of our combined model in predicting the early efficacy of NACT in patients with HER2-positive breast cancer.

There are several limitations to this study. Firstly, the interpretation of breast US characteristics is largely influenced by the sonographer’s personal experience, and the US section images stored in the database may cause the characteristics of other US sections to be ignored; secondly, this study excluded masses that could not be accurately measured by the US, which may result in selective bias; finally, this study was a retrospective, single-center study with a small total sample size. Therefore, a multicenter prospective study with a larger sample size will be conducted in future to further improve and validate.

## Conclusion

In conclusion, US characteristics combined with clinicopathological indices have higher predictive performance than conventional US or clinicopathological indices alone for predicting the early efficacy of HER2-positive breast cancer after NACT. The combined model has improved AUC, sensitivity, specificity, and accuracy, suggesting that our model may provide important decision support for the clinical formulation of HER2-positive breast cancer treatment strategies.

## Data Availability

The datasets used and/or analyzed during the current study are available from the corresponding author on reasonable request.

## Abbreviations

US: Ultrasound
NACT: Neoadjuvant chemotherapy
HER2: Human epidermal growth factor receptor 2
pCR: Pathologic complete response
ROC: Receiver operating characteristic
AUC: Area under the ROC curve
DFS: Disease-free survival
OS: Overall survival
FISH: Fluorescence in situ hybridization
DCIS: Ductal carcinoma in situ
NCCN: National comprehensive cancer network
CACA: China anti-cancer association
SD: Standard deviation (SD)
IDC: Invasive ductal carcinoma
ER: Estrogen receptor
PR: Progesterone receptor
HR: Hormone receptor
NACT: Neoadjuvant chemotherapy
H: Trastuzumab
HP: Trastuzumab plus pertuzumab
PPV: Positive predictive value
NPV: Negative predictive value
FNR: False-negative rate
ASCO: American society of clinical oncology
ADCC: Antibody-dependent cell-mediated cytotoxicity

## References

[1] Sung H, Ferlay J, Siegel RL, Laversanne M, Soerjomataram I, Jemal A (2021). Global cancer statistics 2020: GLOBOCAN estimates of incidence and mortality worldwide for 36 cancers in 185 countries. CA Cancer J Clin.

[2] Zeng X, Liu C, Yao J, Wan H, Wan G, Li Y (2021). Breast cancer stem cells, heterogeneity, targeting therapies and therapeutic implications. Pharmacol Res.

[3] Godone RLN, Leitão GM, Araújo NB, Castelletti CHM, Lima-Filho JL, Martins DBG (2018). Clinical and molecular aspects of breast cancer: targets and therapies. Biomed Pharmacother Biomede Pharmacother.

[4] McDonald ES, Clark AS, Tchou J, Zhang P, Freedman GM (2016). Clinical diagnosis and management of breast cancer. J Nucl Med.

[5] Korde LA, Somerfield MR, Carey LA, Crews JR, Denduluri N, Hwang ES (2021). Neoadjuvant chemotherapy, endocrine therapy, and targeted therapy for breast cancer: ASCO guideline. J Clin Oncol Off J Am Soc Clin Oncol.

[6] Magbanua MJM, Swigart LB, Wu HT, Hirst GL, Yau C, Wolf DM (2021). Circulating tumor DNA in neoadjuvant-treated breast cancer reflects response and survival. Ann Oncol Off J Eur Soc Med Oncol.

[7] Kim R, Chang JM, Lee HB, Lee SH, Kim SY, Kim ES (2019). Predicting axillary response to neoadjuvant chemotherapy: breast MRI and US in patients with node-positive breast cancer. Radiology.

[8] Portnow LH, Kochkodan-Self JM, Maduram A, Barrios M, Onken AM, Hong X (2023). Multimodality imaging review of HER2-positive breast cancer and response to neoadjuvant chemotherapy. Radiogr Rev Publ Radiol Soc N Am Inc.

[9] Untch M, Fasching PA, Konecny GE, Hasmüller S, Lebeau A, Kreienberg R (2011). Pathologic complete response after neoadjuvant chemotherapy plus trastuzumab predicts favorable survival in human epidermal growth factor receptor 2-overexpressing breast cancer: results from the TECHNO trial of the AGO and GBG study groups. J Clin Oncol Off J Am Soc Clin Oncol.

[10] Liu Y, Wang Y, Wang Y, Xie Y, Cui Y, Feng S (2022). Early prediction of treatment response to neoadjuvant chemotherapy based on longitudinal ultrasound images of HER2-positive breast cancer patients by Siamese multi-task network: a multicentre, retrospective cohort study. EClinicalMedicine.

[11] Cortazar P, Zhang L, Untch M, Mehta K, Costantino JP, Wolmark N (2014). Pathological complete response and long-term clinical benefit in breast cancer: the CTNeoBC pooled analysis. Lancet Lond Engl.

[12] Broglio KR, Quintana M, Foster M, Olinger M, McGlothlin A, Berry SM (2016). Association of pathologic complete response to neoadjuvant therapy in HER2-positive breast cancer with long-term outcomes: a meta-analysis. JAMA Oncol.

[13] Pusztai L, Foldi J, Dhawan A, DiGiovanna MP, Mamounas EP (2019). Changing frameworks in treatment sequencing of triple-negative and HER2-positive, early-stage breast cancers. Lancet Oncol.

[14] Rauch GM, Adrada BE, Kuerer HM, van la Parra RFD, Leung JWT, Yang WT (2017). Multimodality imaging for evaluating response to neoadjuvant chemotherapy in breast cancer. Am J Roentgenol.

[15] Cullinane C, Brien AO, Shrestha A, Hanlon EO, Walshe J, Geraghty J (2022). The association between breast density and breast cancer pathological response to neoadjuvant chemotherapy. Breast Cancer Res Treat.

[16] van Ramshorst MS, Loo CE, Groen EJ, Winter-Warnars GH, Wesseling J, van Duijnhoven F (2017). MRI predicts pathologic complete response in HER2-positive breast cancer after neoadjuvant chemotherapy. Breast Cancer Res Treat.

[17] Ochi T, Tsunoda H, Matsuda N, Nozaki F, Suzuki K, Takei H (2021). Accuracy of morphologic change measurements by ultrasound in predicting pathological response to neoadjuvant chemotherapy in triple-negative and HER2-positive breast cancer. Breast Cancer Tokyo Jpn.

[18] Candelaria RP, Bassett RL, Symmans WF, Ramineni M, Moulder SL, Kuerer HM (2017). Performance of mid-treatment breast ultrasound and axillary ultrasound in predicting response to neoadjuvant chemotherapy by breast cancer subtype. Oncologist.

[19] Thomassin-Naggara I, Tardivon A, Chopier J (2014). Standardized diagnosis and reporting of breast cancer. Diagn Interv Imaging.

[20] Adler DD, Carson PL, Rubin JM, Quinn-Reid D (1990). Doppler ultrasound color flow imaging in the study of breast cancer: preliminary findings. Ultrasound Med Biol.

[21] Itoh A, Ueno E, Tohno E, Kamma H, Takahashi H, Shiina T (2006). Breast disease: clinical application of US elastography for diagnosis. Radiology.

[22] Nielsen TO, Leung SCY, Rimm DL, Dodson A, Acs B, Badve S (2021). Assessment of Ki67 in breast cancer: updated recommendations from the international Ki67 in breast cancer working group. J Natl Cancer Inst.

[23] Allison KH, Hammond MEH, Dowsett M, McKernin SE, Carey LA, Fitzgibbons PL (2020). Estrogen and progesterone receptor testing in breast cancer: American Society of Clinical Oncology/College of american pathologists guideline update. Arch Pathol Lab Med.

[24] Loibl S, Gianni L (2017). HER2-positive breast cancer. Lancet Lond Engl.

[25] Ogston KN, Miller ID, Payne S, Hutcheon AW, Sarkar TK, Smith I (2003). A new histological grading system to assess response of breast cancers to primary chemotherapy: prognostic significance and survival. The Breast.

[26] Nahm FS (2022). Receiver operating characteristic curve: overview and practical use for clinicians. Korean J Anesthesiol.

[27] Xu B, Shen J, Zhang L, Zhao W, Wang L (2022). HER2 protein expression level is positively associated with the efficacy of neoadjuvant systemic therapy in HER2-positive breast cancer. Pathol Res Pract.

[28] Ross JS, Slodkowska EA, Symmans WF, Pusztai L, Ravdin PM, Hortobagyi GN (2009). The HER-2 receptor and breast cancer: ten years of targeted anti-HER-2 therapy and personalized medicine. Oncologist.

[29] Giordano SH, Franzoi MAB, Temin S, Anders CK, Chandarlapaty S, Crews JR (2022). Systemic therapy for advanced human epidermal growth factor receptor 2-positive breast cancer: ASCO guideline update. J Clin Oncol Off J Am Soc Clin Oncol.

[30] Kreutzfeldt J, Rozeboom B, Dey N, De P (2020). The trastuzumab era: current and upcoming targeted HER2+ breast cancer therapies. Am J Cancer Res.

[31] Faruki H, Mayhew GM, Serody JS, Hayes DN, Perou CM, Lai-Goldman M (2017). Lung adenocarcinoma and squamous cell carcinoma gene expression subtypes demonstrate significant differences in tumor immune landscape. J Thorac Oncol Off Publ Int Assoc Study Lung Cancer.

[32] Jiang M, Li CL, Luo XM, Chuan ZR, Lv WZ, Li X (2021). Ultrasound-based deep learning radiomics in the assessment of pathological complete response to neoadjuvant chemotherapy in locally advanced breast cancer. Eur J Cancer Oxf Engl.

[33] Hwang HW, Jung H, Hyeon J, Park YH, Ahn JS, Im YH (2019). A nomogram to predict pathologic complete response (pCR) and the value of tumor-infiltrating lymphocytes (TILs) for prediction of response to neoadjuvant chemotherapy (NAC) in breast cancer patients. Breast Cancer Res Treat.

[34] Choi HJ, Ryu JM, Kim I, Nam SJ, Kim SW, Yu J (2019). Nomogram for accurate prediction of breast and axillary pathologic response after neoadjuvant chemotherapy in node positive patients with breast cancer. Ann Surg Treat Res.

[35] Lamb PM, Perry NM, Vinnicombe SJ, Wells CA (2000). Correlation between ultrasound characteristics, mammographic findings and histological grade in patients with invasive ductal carcinoma of the breast. Clin Radiol.

[36] Ring AE, Smith IE, Ashley S, Fulford LG, Lakhani SR (2004). Oestrogen receptor status, pathological complete response and prognosis in patients receiving neoadjuvant chemotherapy for early breast cancer. Br J Cancer.

[37] Kaufmann M, von Minckwitz G, Bear HD, Buzdar A, McGale P, Bonnefoi H (2007). Recommendations from an international expert panel on the use of neoadjuvant (primary) systemic treatment of operable breast cancer: new perspectives 2006. Ann Oncol Off J Eur Soc Med Oncol.

[38] De Nunzio MC, Evans AJ, Pinder SE, Davidson I, Wilson ARM, Yeoman LJ (1997). Correlations between the mammographic features of screen detected invasive breast cancer and pathological prognostic factors. The Breast.

[39] Mazari FAK, Sharma N, Dodwell D, Horgan K (2018). Human epidermal growth factor 2-positive breast cancer with mammographic microcalcification: relationship to pathologic complete response after neoadjuvant chemotherapy. Radiology.

[40] Baumgartner A, Tausch C, Hosch S, Papassotiropoulos B, Varga Z, Rageth C (2018). Ultrasound-based prediction of pathologic response to neoadjuvant chemotherapy in breast cancer patients. Breast Edinb Scotl.

[41] Dobruch-Sobczak K, Piotrzkowska-Wróblewska H, Klimonda Z, Roszkowska-Purska K, Litniewski J (2019). Ultrasound echogenicity reveals the response of breast cancer to chemotherapy. Clin Imaging.

[42] Cui H, Zhao D, Han P, Zhang X, Fan W, Zuo X (2021). Predicting pathological complete response after neoadjuvant chemotherapy in advanced breast cancer by ultrasound and clinicopathological features using a nomogram. Front Oncol.

